# Methodical inaccuracy of the Z-scan method for few-cycle terahertz pulses

**DOI:** 10.1038/s41598-019-45735-6

**Published:** 2019-06-24

**Authors:** Maksim Melnik, Irina Vorontsova, Sergey Putilin, Anton Tcypkin, Sergei Kozlov

**Affiliations:** ITMO University, International Laboratory of Femtosecond Optics and Femtotechnologies, St. Petersburg, 197101 Russia

**Keywords:** Terahertz optics, Nonlinear optics

## Abstract

Modern sources of THz radiation generate high-intensity pulses allowing to observe nonlinear effects in this spectral range. To describe many nonlinear effects theoretically, it is necessary to know the nonlinear refractive index coefficient of optical materials. The work studies the applicability of the Z-scan method to determine the nonlinear refractive index coefficient in the THz frequency range for few-cycle pulses. We have discussed the correctness of the known Z-scan method for calculating the nonlinear refractive index coefficient for broadband THz radiation regarding number of cycles pulses have. We have demonstrated that the error in determining the nonlinear refractive index coefficient is always greater than 70% for true single-cycle pulses. With the increase in the number of oscillations to the measurement error shows strong dependence on the sample thickness and can vary from 2% to 90% regarding the parameters chosen. The fact that such radiation dispersion length is commensurate with the nonlinear length or even less than the latter results in the discrepancy mentioned. It is demonstrated that the decrease in the sample thickness leads to the reduction of the nonlinear refractive index coefficient determination error, and this error is <2% when the ratio between the sample thickness and the pulse longitudinal spatial size is ≤1. This can relate to the fact that the nonlinear effects in such a thin sample occur faster than the dispersion ones.

## Introduction

Currently, the study of the terahertz (THz) frequency range of optical radiation is one of the developing and promising scientific fields. The reason for active performing in the area lies in the broad applications of THz radiation in biology^1,2^, medicine^3,4^, security systems^5^, non-destructive evaluation^6,7^, wireless information transmission^8^. Until recently practical devices and systems were mainly developed using linear effects of THz wave optics^9^. At present, there are sources of pulsed THz radiation with energy in a single pulse up to 1 mJ have appeared^10,11^, i.e., the peak values of the electric field can reach values up to hundreds of MV/cm^12^. This allowed to move to profound research in the field of nonlinear THz optics^13–15^. High-energy sources were constructed using singe-and-double-color filamentation^16–18^, as well as using femtosecond pulses rectification in LiNbO_3_ crystals^10,11,14,19^.

First experimental observations of such a classical nonlinear phenomenon as self-phase modulation in THz nonlinear optics^20^ demonstrated significant changes in the refractive index of materials. The theoretical work^21^ predicts that values of the fast-response nonlinear refractive index coefficient (*n*_2_) of materials in the THz spectral range are several orders of magnitude higher than its values for ultrashort pulses of the visible and near-IR spectral ranges. Moreover, nonlinear effects are experimentally observed in semiconductors^22,23^ in the THz frequency range, for which the theory was preliminarily built^24,25^ using nonlinear THz optics. Regarding rotational states of gas molecules, it was shown, both experimentally and theoretically^26,27^, that the coherent control of THz radiation by populations of rotational sublevels and their relaxation is possible. The work^28^ investigated nonlinear four-wave mixing in gases between intense ultrashort optical pulses and THz fields.

There are several techniques for measuring *n*_2_ in IR and optical spectral ranges. One of the most common ones is the Z-scan method^29^. Used for monochromatic radiation, the technique can also be applied to femtosecond radiation, which radiation spectrum is quite wide^30^. Recently, the first works on preliminary experiments estimating *n*_2_ coefficient using the Z-scan technique in THz spectral range clearly showed the presence of a significant nonlinear effect^31,32^. However, the application of the Z-scan method raises several questions since the spectrum of pulsed THz sources is even wider than femtosecond one^33^ and its electric field can have few cycles only^34,35^. Thus, in the experimental works mentioned estimates of the nonlinear refractive index coefficient were made only, while for a precise determination of the latter it is necessary to reveal the influence of the pulse cycle number on the applicability of the Z-scan method.

This work is devoted to the theoretical study of the Z-scan method applicability for evaluating *n*_2_ coefficient of isotropic transparent materials in the field of broadband few-cycle THz pulses. A comparative analysis was conducted with the standard analytical model of this method for monochromatic radiation. As a result, it is shown that for non-single-cycle pulses, the Z-scan for broadband THz radiation correlates with the analytics of this method for monochromatic radiation well. In the case of three and less cycle broadband THz pulses, an error of *n*_2_ estimation appears. The latter has a constanly high value for true single-cycle pulses and depends on the ratio of the sample thickness and spatial size of THz pulse for 1.5–3 cycle ones.

## Results

To analyze the applicability of the Z-scan method for broadband few-cycle THz pulses the results of the numerical simulation were compared with the analytical model of the method for monochromatic radiation (see Methods). It should be mentioned that the pulse duration does not appear in the equations, and therefore, the analytical curve does not depend on this pulse characteristic. The numerical simulation of the Z-scan method was conducted using the equations of intense light pulse propagation in a dielectric medium with normal group dispersion and nonresonant nonlinearity^36^ (see Methods). Using this model the propagation of a spherical THz beam at two focal lengths was numerically simulated. For different positions of the nonlinear medium sample on the optical axis of propagation, the resulting THz pulses were detected through the aperture.

**Figure 1:**
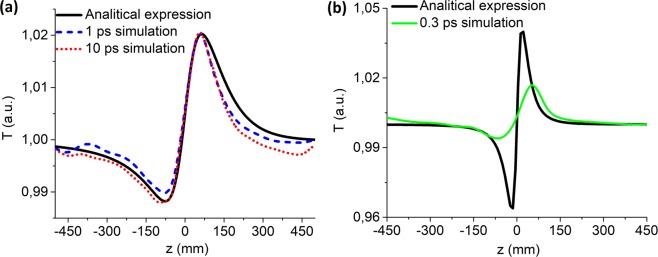
Z-scan curves (colored) obtained through the numerical simulation of the method (see Methods) for (**a**) 1 and 10*T*_0_ and (**b**) 0.3*T*_0_ pulses for the central wavelength *λ*_0_ = 0.3 mm and crystal thickness of 0.3 mm; black curve corresponds to the analytical model for monochromatic THz radiation.

Figure 1 shows the results of the numerical simulation of the typical Z-scan method curves (see Methods). They represent the dependence of the transmission through the closed aperture *T* on the sample position *z* for the following parameters: central wavelength of the THz radiation *λ* = 0.3 mm, period of oscillation *T*_0_ = 1 ps, peak intensity in the caustic (a) *I*_0_ = 3.1 ⋅ 10^8^ W/cm^2^, and (b) *I*_0_ = 8.3 ⋅ 10^8^ W/cm^2^, duration*τ*_0_ = 0.3, 1, 10*T*_0_, thickness of the ZnSe *L* = 0.3 mm. The value of the nonlinear refractive index coefficient *n*_2_ = 4 ⋅ 10^−11^ cm^2^/W^31^. As seen, the obtained curves in Fig. 1 qualitatively correspond to the Z-scan curves. For the central wavelength *λ*_0_ = 0.3 mm the duration *τ* = 0.3 ps corresponds to the single oscillation of electromagnetic field^37^. In the simulation in Fig. 1(b) a different intensity value is used due to the fact that in the case of a true single-cycle pulse its waist diameter is 2 times less than the one of multi-cycle pulses and the focusing of such a pulse leads to its transformation into a 1.5-cycle pulse^37^. It happens since the central frequency shifts to higher values (see Methods) in the case of a true single-cycle pulse.

Noteworthy, the shorter the pulse duration is the less its peak-to-valley ratio corresponds to the one the analytical curve has, and therefore, the higher the inaccuracy of the obtained results of *n*_2_ estimations is. It can be concluded that the Z-scan method introduces the most significant inaccuracy in the determination of *n*_2_ when it comes to pulses of less than 2 oscillations. As mentioned above, for a true single-cycle pulse a change in its duration in the caustic and decrease in waist size are observed. Additionally, a shift of the central frequency to the higher values occures. All the facts mentioned result in the peak intensity in the caustic increase by 2.7 times (see Methods). Despite this fact, the peak-to-valley ratio of the latter is smaller than even for a two-cycle pulse, as seen in Fig. 1. The analytical expression gives the value of 4.0 ⋅ 10^−11^ cm^2^/W for *n*_2_, whereas the numerical simulation curve leads to the value of 1.15 ⋅ 10^−11^ cm^2^/W. Thus, the inaccuracy of the method, in this case, is more than 70%.

**Figure 2:**
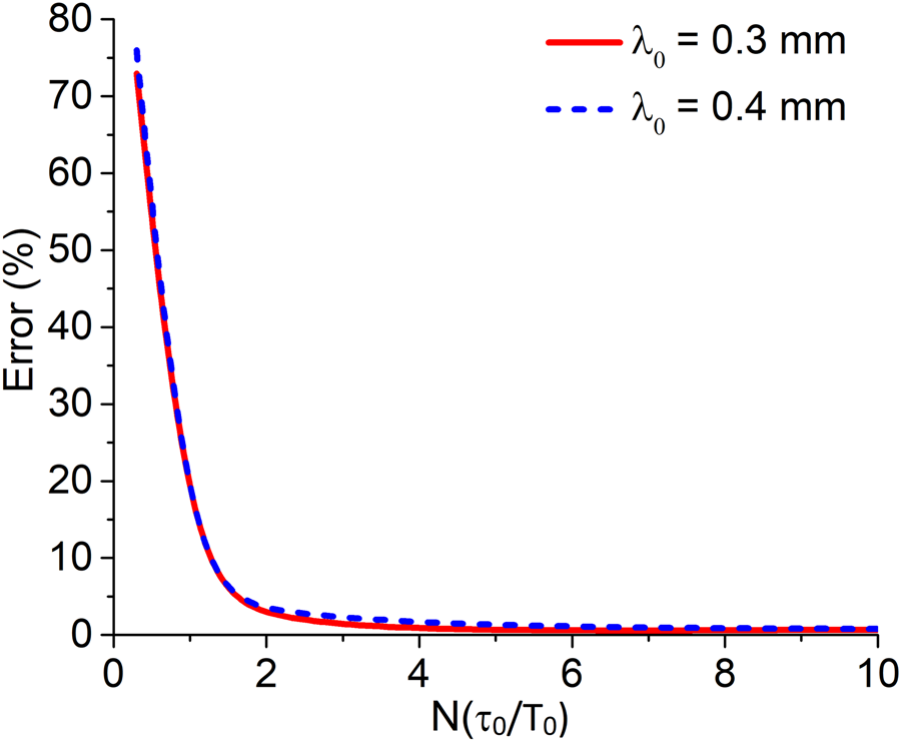
Dependence of *n*_2_ measurement error on the number of THz pulse periods for a fixed thickness of the sample *L* = 0.3 mm and pulse wavelengths *λ*_0_ = 0.3 mm and 0.4 mm.

Figure 2 illustrates the dependence of the magnitude of the error of *n*_2_ calculation from the differential curve obtained by numerical simulation of the Z-scan method on the number of cycles of the THz pulse *T*_0_ in the case of a fixed sample thickness *L* = 0.3 mm for different values of the wavelength *λ*_0_ = 0.3 mm and 0.4 mm.

The results show that with a decrease in the number of pulse cycles when the pulse has less than 2 oscillations, the magnitude of *n*_2_ measurement error crucially increases. The highest magnitude of the error corresponds to a true single-cycle pulse. As seen, the magnitude of the error practically does not change for different wavelengths.

## Discussion

**Figure 3:**
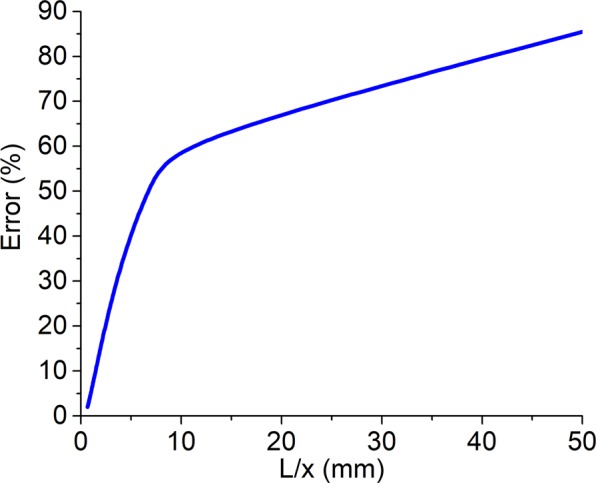
The dependence of *n*_2_ measurement error on the value of the L/x ratio.

The reason for the discrepancy between the simulation results for pulses with less than 2 oscillations and the analytical calculations for monochromatic THz radiation may result from the fact that the dispersion effects on such a pulse are consistent with the nonlinearity^38^. For the few-cycle pulses, the dispersion length *L*_*disp*_ commensurates the nonlinear length *L*_*nl*_ or even less than the latter (see Methods)^38^. The calculation of the dispersion and the nonlinear lengths for few-cycle pulses was performed (see Methods). For example, in the case of the true single-cycle pulse with the central wavelength *λ*_0_ = 0.3 mm they are *L*_*disp*_ = 7.81 mm and *L*_*nl*_ = 9.07 mm correspondingly. These values are larger than the sample thickness *L* = 0.3 mm. We assumed that an increase in the sample thickness would lead to a larger error in *n*_2_ estimation while its decrease would lead to the smaller one. The latter can be related to thinning of the sample which causes the nonlinear properties of the medium to occur faster than the dispersive ones. To confirm this assumption, we performed a simulation for different wavelengths *λ*_0_ = 0.2–0.6 mm and different sample thicknesses *L* = 0.06–4.5 mm. To colligate the results for all values of central wavelengths, the general parameter *L*/*x* was introduced. It is the ratio of the sample thickness *L* to the spatial size of the pulse and describes the number of pulses fitting inside the sample, was introduced. The dependence of the magnitude of *n*_2_ measurement error on the ratio between the sample thickness and the spatial size of the pulse *L*/*x* is the same for all the wavelengths and is shown in Fig. 3.

It is seen that for broadband THz single-cycle pulsed radiation, the magnitude of *n*_2_ measurement by the Z-scan method error increases with the *L*/*x* ratio. For the sample thickness corresponding to the *L*/*x* ratio less than 10, the rapid increase in *n*_2_ measurement error is observed. For *L*/*x* = 10 *n*_2_ measurement error is equal to more than 70%. Otherwise, when *L*/*x* > 10, the slope of the curve is smaller and approaches the values close to the saturation (90% approximately). Accordingly, the most accurate results are achieved in the case of the lowest value of the last relation. Since the size of the sample cannot be infinitely reduced, the best option is when the ratio $L/x\le 1$ and the Z-scan method has a negligibly small error of *n*_2_ measurement. This may be explained by the fact that nonlinear effects occur faster than the dispersion one.

## Methods

### Analytical model of the Z-scan method for quasi-monochromatic radiation

The work^29^ proposes to calculate the Z-scan curve differential (the transmission through the closed aperture *T*) using the following formula:

1 $$T(z)=\frac{{\int }_{-\infty }^{+\infty }\,{P}_{T}({{\rm{\Delta }}{\rm{\Phi }}}_{0}(t))dt}{S\cdot {\int }_{-\infty }^{+\infty }\,{P}_{i}(t)dt},$$

where ${P}_{i}(t)=\frac{\pi {w}_{0}^{2}{I}_{0}(t)}{2}$ is the instantaneous input power (within the sample), $S=1-\exp (-2\frac{{r}_{a}^{2}}{{\omega }_{a}^{2}})$ is the aperture linear transmittance; the transmitted power through the aperture gives

2 $${P}_{T}({{\rm{\Delta }}{\rm{\Phi }}}_{0}(t))=c{\varepsilon }_{0}{N}_{0}\pi {\int }_{0}^{{r}_{a}}\,a|{E}_{a}(r,t){|}^{2}rdr,$$

where $${E}_{a}(r,t)=E(z,r=0,t)\cdot exp(-\frac{\alpha L}{2})\cdot \sum _{m=0}^{+\infty }\frac{{[i{\rm{\Delta }}{\phi }_{0}(z,t)]}^{m}}{m!}\frac{{w}_{{m}_{o}}}{{w}_{m}}\cdot exp(-\frac{{r}^{2}}{{w}_{m}^{2}}-\frac{ik{r}^{2}}{2{R}_{m}}+i{Q}_{m})$$, $$E(z,r=0,t)=$$$${E}_{0}\,\sin (2\pi {\nu }_{0}t)\frac{{w}_{o}}{w(z)}$$, $${\rm{\Delta }}{\phi }_{0}(z,t)=\frac{{{\rm{\Delta }}{\rm{\Phi }}}_{0}(t)}{1+\frac{{z}^{2}}{{z}_{0}^{2}}}$$, ΔΦ_0_(*t*) = *k*Δ*n*_0_(*t*)*L*.

The following values are used in these formulas: absorption coefficient *α* = 0.85 cm^−1^, sample length *L* = 0.3 mm, central frequency of the radiation *ν*_0_ = 1 THz (*λ*_0_ = 0.3 mm), beam waist radius *w*_0_ = 1.4 mm, aperture radius *r*_*a*_ = 1.5 mm, radius of the THz beam *w*_*a*_ = 12.5 mm, intensity of the THz beam in caustic *I*_0_ = 3 ⋅ 10^8^ W/cm^2^, nonlinear refractive index coefficient *n*_2_ = 4 ⋅ 10^−11^ cm^2^/W. This value of *n*_2_ was obtained in experiment previously^31^.

### Numerical simulation of the Z-scan method

In the paper^21^ analytical model for calculating the non-resonant vibrational contribution to the nonlinear refractive index *n*_2_ (Kerr coefficient) is developed. It is demonstrated that the value of this contribution in the THz spectral region can exceed the value of *n*_2_ in the visible and near-IR spectral ranges by several orders of magnitude. Also, authors show that for the case of ultrashort optical pulses, including intense picosecond THz pulses, the dominant source of nonlinearity tends to be the low-inertia ones where nonlinear mechanisms are based on the nonlinear response of each molecule to the radiation field. Noteworthy, for the pulses in the THz range, one expects the dominant mechanism of nonlinearity to be associated with anharmonic vibrations of the lattice, unlike visible and IR, where nonlinearities has the electronic nature. These mechanisms are well-described with nonlinear refractive index coefficient *n*_2_ as a material characteristic. These findings have now been confirmed experimentally^31,39,40^. Thus, the use of the Z-scan method is justified allowing to calculate the Kerr nonlinearity in the THz frequency range. Work^21^ shows that *n*_2_ dispersion of the vibrational nature of crystals can usually be neglected in the THz frequency range. *n*_2_ dispersion can significantly increase in the region of two-photon resonance with the vibrational mode of the crystal or natural oscillation of the molecule in liquid. For example, in water, oscillations that determine its vibrational nonlinearity in the far IR range of the spectrum have frequency of 15.9 THz while central frequency of used THz radiation is 0.75 THz^39^. Accordingly, up to the frequency values of 8 THz a small dispersion of a vibrational nature in water is expected. Therefore, in our work, we assume that *n*_2_ is considered as a constant value.

**Figure 4:**
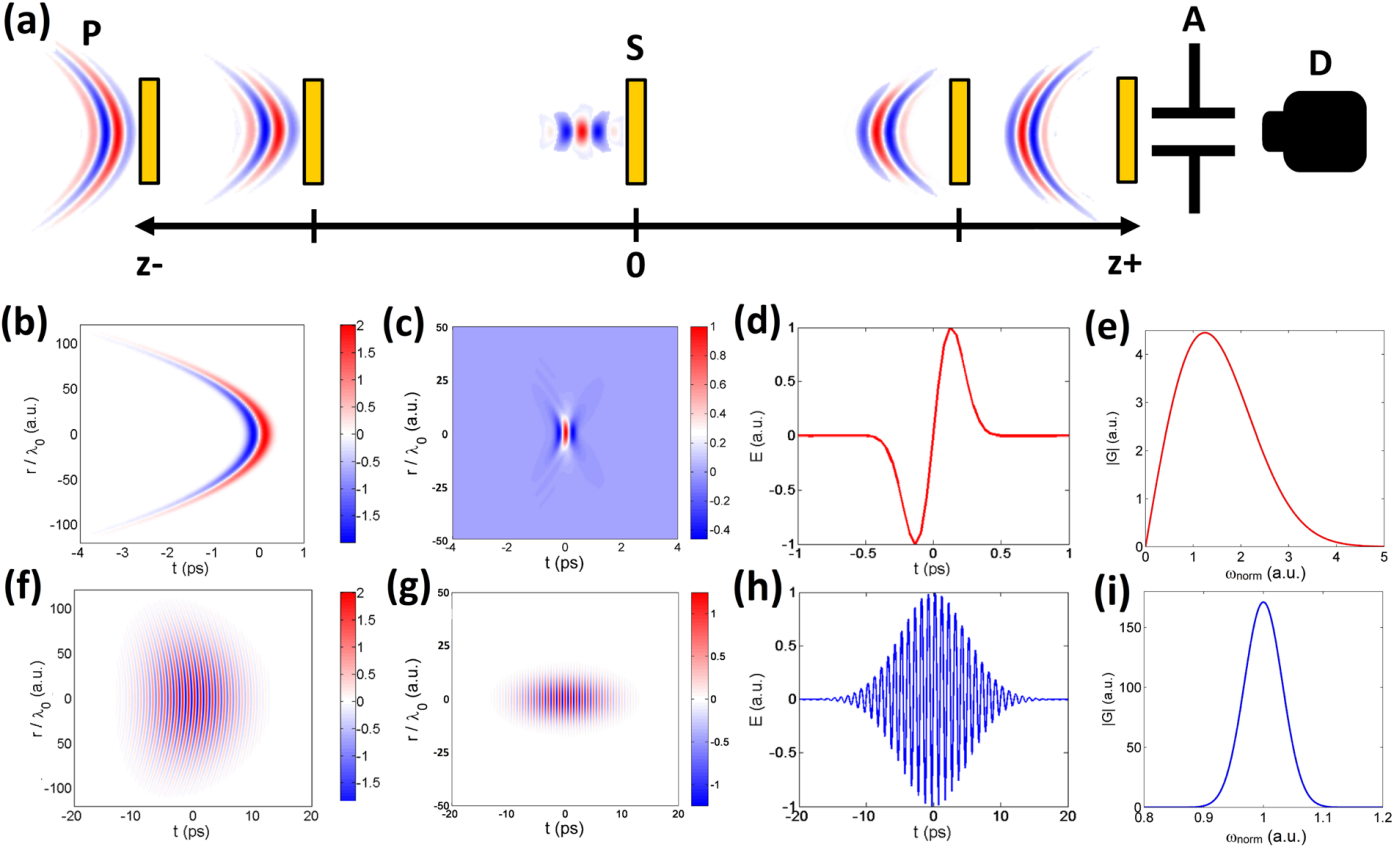
(**a**) Visual representation of the Z-scan method for the THz pulsed radiation propagating at the distance from z− to z+: for each position of the crystal the THz pulse was distributed through the air, the crystal itself and then further through the remaining distance in air to be finally captured at the detector, placed at z+. The distance of propagation corresponds to two focal lengths. Single-cycle (**b**,**c**) and multi-cycle (**f**,**g**) THz pulses representation at the input and in the focus correspondingly; (**d**,**h**) cross-sections of their electric field profiles correspondingly; (**e**,**i**) their spectra.

It is well known that the essence of the Z-scan method consists of induced narrowing and expansion of an intense spherical light beam when a nonlinear medium moves along the axis of its propagation and passes through the focus^29^. In this case, the nonlinear medium plays the role of a thin lens and its placement in or near the focus leads to a minimal change in the distribution of the beam field in the far field. The resulting characteristic Z-scan curve represents the peak and valley of the transmission of the nonlinear medium, from the magnitude of their difference it is possible to determine the value of the nonlinear refractive index coefficient. In this work, we performed a numerical simulation of the Z-scan method, an illustration of which is shown in Fig. 4(a). It illustrates the propagation of a spherical THz beam at two focal lengths. In this graphical representation, the minimum of the electric field corresponds to the color of blue, and the maximum of the electric field to red. For each position of the ZnSe crystal, the THz pulse was distributed through the air, the crystal itself and then further through the remaining distance in the air to be finally captured at the detector through the aperture.

Figure 4(b–i) represents examples of single and multi-cycle pulses and a central wavelength of 300 *μ*m. To represent the transverse size of pulse fractions of wavelengths calculated as *r*/*λ*_0_ ratio are used as units. A pulse with a duration of 1 ps, in fact, has 1.5 cycles, whereas a true single-cycle pulse has a duration of 0.3 ps. It can be seen in Fig. 4(d) though, that the spectrum of a true single-cycle pulse shifts to the higher value area. Regarding the numerical simulation performed, this fact means that the central wavelength of the radiation becomes smaller and, therefore, the waist radius in the caustic also decreases. In turn, this leads to the peak intensity in the caustic raise by 1.5 times and should cause an increase in the peak-to-valley ratio of the Z-scan curve. A true single-cycle pulse corresponds to the ideal THz pulse^37^. However, generated THz pulses have the form of the first type in real experiments^41^. It should be noted that despite the focal length being much larger than the transverse size of the THz field (which is consistent with the case of the paraxial propagation), a very strong curvature of the wavefront takes place for a true single-cycle pulse. The latest analysis of nonlinear effects for nonparaxial beams of single-cycle THz pulses^42^ shows that the paraxial approximation characterizes the dynamics of the transverse field component and strongly focused nonparaxial THz beams well. The appearance and nontrivial dynamics of the longitudinal field component are the difference in this case.

The numerical simulation of the Z-scan method was conducted for the following system parameters: sample under the investigation - ZnSe (its thickness *L* = 0.06–0.3 mm), focal length of the lens *f* = 50 cm, central wavelength of the THz pulse *λ*_0_ = 0.2–0.6 mm, with period of oscilation *T*_0_ = 0.75–2 ps, pulse duration *δt* = 0.3–10*T*_0_, the transverse width of the input beam *D* = 25 mm, size of the aperture *A* = 1.5 mm, the peak intensity in the caustic *I*_0_ = 3.1 ⋅ 10^8^ W/cm^2^. The dispersion parameters are specified in^43^ and are approximated by the formula *n*_0_(*ω*) = *N*_0_ + *acω*^2^, where *N*_0_ = 3 is the refractive index at the “zero” frequency, and *a* = 6 ⋅ 10^−36^ s^3^/m is the dispersion coefficient; *n*_2_ value is taken from the paper^31^ as the result of experimental data evaluation and is equal to *n*_2_ = 4 ⋅ 10^−11^ cm^2^/W. To simulate the propagation of THz pulse in the air and ZnSe we use the equations of an intense light pulse propagation in a waveguide dielectric medium with a normal group dispersion and nonresonant nonlinearity^36^:

3 $$\frac{\partial E}{\partial z}-a\frac{{\partial }^{3}E}{\partial {\tau }^{3}}+g{E}^{2}\frac{\partial E}{\partial \tau }=\frac{c}{2{N}_{0}}{{\rm{\Delta }}}_{\perp }{\int }_{-\infty }^{\tau }Ed\tau ^{\prime} ,$$

where the second item from the left part describes the dispersion of the linear polarization response of the electronic and vibrational nature, the third one describes the nonlinearity of the response of the electron nature, and the term in the right part describes the diffraction of the extremely short pulse. In this equation, *z* is the sample position measured with respect to the focal plane, Δ_⊥_ - Transverse Laplacian, *τ* = *t* − *n*_0_*z*/*c* – time in the accompanying coordinate system, $E=E(z=0,r,t)={E}_{0}\cdot \exp (-\frac{{r}^{2}}{{r}_{0}^{2}})\cdot \exp (-\frac{{t}^{2}}{{\tau }_{0}^{2}})\cdot \,\sin \,{\omega }_{0}t\cdot R(x,y)$ – the initial spherical electric field. Here $r=\sqrt{{x}^{2}+{y}^{2}}$ is the radial coordinate, ${r}_{0}=\frac{d}{2}$ is the transverse radius of the input beam, *R*(*x*, *y*) = exp(−*ik*(*x*^2^ + *y*^2^)/2*f*) is the transmission function of the spherical lens with the focal length *f*, *k* = *n*_0_*ω*/*c* is the wavenumber, *x* and *y* are the transversed coordinates.

During the data processing of an array of numbers characterizing the detected signal at each crystal position, the energy was integrated along the time axis for an aperture with the radius of *r*_0_ = 0.75 mm and *t* = 2000 fs using the equation:

4 $${\int }_{r=0}^{r={r}_{0}}\,{\int }_{\phi =0}^{\phi =2\pi }\,{\int }_{\tau =-t}^{\tau =t}\,{E}^{2}(\tau ,\phi ,r)d\tau d\phi dr.$$

It should be noted that few-cycle THz pulses are critically short regarding the number of oscillations. As known, the envelope approach was historically suggested as a method to analyze the evolution of pulses containing a large number of field cycles, therefore, to precisely describe the dynamics of few-cycle pulses additional equations are needed to be introduced. The latter leads to the mathematical model be more cumbersome. The field approach, in turn, allows to express all the necessary information about a pulse, including generation at triple and other high frequencies, in one equation only^36^. Therefore, the key problem of the theoretical study of their propagation laws in a nonlinear media is the need to improve existing^36^ and elaborate new approaches to the analysis of the field dynamics and emission spectra associated with extremely short THz pulses features.

### The dispersion and the nonlinear lengths calculation

To perform the calculation of the dispersion and nonlinear lengths for the few-cycle pulses the following formulas have been used^37^:

5 $${L}_{disp}=\frac{{\pi }^{2}{\lambda }_{0}^{2}{N}_{0}}{16{\rm{\Delta }}{n}_{disp}},$$

6 $${L}_{nl}=\frac{{\lambda }_{0}^{2}{N}_{0}}{16{\rm{\Delta }}{n}_{nl}},$$

where ${\rm{\Delta }}{n}_{disp}=ac{\omega }_{0}^{2}$ is a modification of the refractive index at the central wavelength *λ*_0_ due to dispersion, ${\rm{\Delta }}{n}_{nl}=\frac{1}{2}\cdot {n}_{2}I$ is a nonlinearly induced change of the optical refractive index, ${\omega }_{0}=\frac{2\pi }{{T}_{0}}$ is the central optical frequency, and *N*_0_ is the refractive index at the “zero” frequency, *a* is the dispersion coefficient, which describe the dispersive properties of the medium: *n*_0_(*ω*) = *N*_0_ + *acω*^2^.

Therefore, for *N*_0_ = 3, *a* = 6 ⋅ 10^−36^ s^3^/m, *λ*_0_ = 0.3 mm (*ω*_0_ = 6.28 ⋅ 10^12^ rad/s, *I*_0_ = 3.1 ⋅ 10^8^ W/cm^2^, and *n*_2_ = 4 ⋅ 10^−11^ cm^2^/W the dispersion and the nonlinear lengths are *L*_*disp*_ = 7.81 mm and *L*_*nl*_ = 9.07 mm correspondingly.

## Conclusion

The work has studied the Z-scan method applicability for the broadband pulsed THz radiation of various number of the field oscillations through the numerical simulation. Numerical Z-scan curves for broadband THz radiation of three and more cycles show good correspondence with the analytical curve for monochromatic THz radiation, while for true single-cycle pulses *n*_2_ measurement error occurs constantly and is always high - 70% approximately. Furthermore, regardless of the central wavelength of the radiation, an increase in the error of *n*_2_ measurement with an increase in the sample thickness is observed for pulses with 1.5–3 cycles. *n*_2_ measurement error can be as large as 90% in this case. This results from the fact that for such radiation the dispersion length is comparable or even less than the nonlinear length. Meanwhile, *n*_2_ measurement error decreases along with the sample thickness. As a result, it has been shown that an essential parameter of the correctness of *n*_2_ measurement for few-cycle pulses is the ratio *L*/*x*, where *L* is the sample thickness, and *x* is the spatial size of the pulse. It was found that the magnitude of the error increases together with the magnitude of the ratio. For *L*/*x* < 1 *n*_2_ measurement error equals to less than 2%. Therefore, it is recommended to use samples which thickness does not exceed the longitudinal spatial size of the pulse to measure its nonlinear parameters using the Z-scan method. This is explained by the fact that the nonlinear effects occur faster than the dispersive ones in such a thin sample.

This study is useful for working in the field of nonlinear optics and using the Z-scan method to determine various nonlinear parameters of media in different spectral ranges.

## References

[1] Lee Soonsung, Kang Hyeona, Do Youngwoong, Lee Gyuseok, Kim Jinwoo, Han Haewook (2016). High-precision THz Dielectric Spectroscopy of Tris-HCl Buffer. Journal of the Optical Society of Korea.

[2] Yamazaki S (2018). Actin polymerization is activated by terahertz irradiation. Sci. Reports.

[3] Smolyanskaya O. A., Schelkanova I. J., Kulya M. S., Odlyanitskiy E. L., Goryachev I. S., Tcypkin A. N., Grachev Ya. V., Toropova Ya. G., Tuchin V. V. (2018). Glycerol dehydration of native and diabetic animal tissues studied by THz-TDS and NMR methods. Biomedical Optics Express.

[4] Smirnov S. V., Grachev Ya. V., Tsypkin A. N., Bespalov V. G. (2014). Experimental studies of the possibilities of diagnosing caries in the solid tissues of a tooth by means of terahertz radiation. Journal of Optical Technology.

[5] Cooper K, Appleby R (2017). Terahertz components and systems for defence and security imaging. J. Phys. D: Appl. Phys.

[6] Devi, N., Dash, J., Ray, S. & Pesala, B. Non-invasive characterization of carbon fiber reinforced polymer composites using continuous wave terahertz system. 1–4, 10.1109/TIMA.2017.8064807 (2017).

[7] Dong, J., Locquet, A., Melis, M. & Citrin, D. S. Global mapping of stratigraphy of an old-master painting using sparsity-based terahertz reflectometry. *Sci. Reports* 7, 10.1038/s41598-017-15069-2 (2017). 10.1038/s41598-017-15069-2 PMC5678175 29118333

[8] Grachev YV (2018). Wireless data transmission method using pulsed THz sliced spectral supercontinuum. IEEE Photonics Technol. Lett..

[9] Bratman VL, Litvak AG, Suvorov EV (2011). Mastering the terahertz domain: sources and applications. Physics-Uspekhi.

[10] Sell A, Leitenstorfer A, Huber R (2008). Phase-locked generation and field-resolved detection of widely tunable terahertz pulses with amplitudes exceeding 100 MV/cm. Opt. Lett..

[11] Junginger F (2010). Single-cycle multiterahertz transients with peak fields above 10 MV/cm. Opt. Lett..

[12] Shalaby, M. & Hauri, C. P. Demonstration of a low-frequency three-dimensional terahertz bullet with extreme brightness. *Nat. Commun*. **6**, 10.1038/ncomms6976 (2015). 10.1038/ncomms6976 25591665

[13] Jones RR, You D, Bucksbaum PH (1993). Ionization of rydberg atoms by subpicosecond half-cycle electromagnetic pulses. Phys. Rev. Lett..

[14] Hebling J, Yeh K-L, Hoffmann MC, Bartal B, Nelson KA (2008). Generation of high-power terahertz pulses by tiltedpulse-front excitation and their application possibilities. J. Opt. Soc. Am. B.

[15] Junginger, F. *et al*. Nonperturbative interband response of a bulk InSb semiconductor driven off resonantly by terahertz electromagnetic few-cycle pulses. *Phys. Rev. Lett*. **109**, 10.1103/physrevlett.109.147403 (2012). 10.1103/PhysRevLett.109.147403 23083284

[16] Bartel T, Gaal P, Reimann K, Woerner M, Elsaesser T (2005). Generation of single-cycle THz transients with high electric-field amplitudes. Opt. Lett..

[17] Kim K-Y, Glownia JH, Taylor AJ, Rodriguez G (2007). Terahertz emission from ultrafast ionizing air in symmetry-broken laser fields. Opt. Express.

[18] Oh TI (2013). Intense terahertz generation in two-color laser filamentation: energy scaling with terawatt laser systems. New J. Phys..

[19] Hirori H, Doi A, Blanchard F, Tanaka K (2011). Single-cycle terahertz pulses with amplitudes exceeding 1 MV/cm generated by optical rectification in LiNbO3. Appl. Phys. Lett..

[20] Turchinovich, D., Hvam, J. M. & Hoffmann, M. C. Self-phase modulation of a single-cycle terahertz pulse by nonlinear free-carrier response in a semiconductor. *Phys. Rev. B* **85**, 10.1103/physrevb.85.201304 (2012).

[21] Dolgaleva, K., Materikina, D. V., Boyd, R. W. & Kozlov, S. A. Prediction of an extremely large nonlinear refractive index for crystals at terahertz frequencies. *Phys. Rev. A* **92**, 10.1103/physreva.92.023809 (2015).

[22] Grishunin K (2019). Transient second harmonic generation induced by single cycle thz pulses in ba 0.8 sr 0.2 tio 3/mgo. Sci. reports.

[23] Knighton BE (2019). Terahertz waveform considerations for nonlinearly driving lattice vibrations. J. Appl. Phys..

[24] Shimano R, Kuwata-Gonokami M (1994). Observation of autler-townes splitting of biexcitons in CuCl. Phys. Rev. Lett..

[25] Citrin D (2001). Toward a semiconductor-based terahertz nonlinear medium. Phys. E: Low-dimensional Syst. Nanostructures.

[26] Fleischer, S., Zhou, Y., Field, R. W. & Nelson, K. A. Molecular orientation and alignment by intense single-cycle THz pulses. *Phys. Rev. Lett*. **107**, 10.1103/physrevlett.107.163603 (2011). 10.1103/PhysRevLett.107.163603 22107382

[27] Fleischer, S., Field, R. W. & Nelson, K. A. Commensurate two-quantum coherences induced by time-delayed THz fields. *Phys. Rev. Lett*. **109**, 10.1103/physrevlett.109.123603 (2012). 10.1103/PhysRevLett.109.123603 23005948

[28] Clerici M (2013). Spectrally resolved wave-mixing between near- and far-infrared pulses in gas. New J. Phys..

[29] Sheik-Bahae M, Said A, Wei T-H, Hagan D, Stryland EV (1990). Sensitive measurement of optical nonlinearities using a single beam. IEEE J. Quantum Electron..

[30] Zheng X (2015). Characterization of nonlinear properties of black phosphorus nanoplatelets with femtosecond pulsed z-scan measurements. Opt. Lett..

[31] Tcypkin A (2018). Experimental estimate of the nonlinear refractive index of crystalline znse in the terahertz spectral range. Bull. Russ. Acad. Sci. Phys..

[32] Woldegeorgis A (2018). THz induced nonlinear effects in materials at intensities above 26 GW/cm2. J. Infrared, Millimeter, Terahertz Waves.

[33] Cao H, Linke RA, Nahata A (2004). Broadband generation of terahertz radiation in a waveguide. Opt. Lett..

[34] Yeh K-L, Hebling J, Hoffmann MC, Nelson KA (2008). Generation of high average power 1 khz shaped THz pulses via optical rectification. Opt. Commun..

[35] Shen, Y. *et al*. Nonlinear cross-phase modulation with intense single-cycle terahertz pulses. *Phys. Rev. Lett*. **99**, 10.1103/physrevlett.99.043901 (2007). 10.1103/PhysRevLett.99.043901 17678365

[36] Kozlov, S. A. & Samartsev, V. V. Fundamentals of Femtosecond Optics (Woodhead Publishing Series in Electronic and Optical Materials Book 52), https://www.amazon.com/Fundamentals-Femtosecond-Publishing-Electronic-Materials-ebook/dp/B00H1YWFHY?SubscriptionId=AKIAIOBINVZYXZQZ2U3A&tag=chimbori05-20&linkCode=xm2&camp=2025&creative=165953&creativeASIN=B00H1YWFHY (Woodhead Publishing, 2013).

[37] Drozdov, A. A., Kozlov, S. A., Sukhorukov, A. A. & Kivshar, Y. S. Self-phase modulation and frequency generation with few-cycle optical pulses in nonlinear dispersive media. *Phys. Rev. A* **86**, 10.1103/physreva.86.053822 (2012).

[38] Kozlov SA (2018). Self-focusing does not occur for few-cycle pulses. J. Physics: Conf. Ser..

[39] Tcypkin Anton N., Melnik Maksim V., Zhukova Maria O., Vorontsova Irina O., Putilin Sergey E., Kozlov Sergei A., Zhang Xi-Cheng (2019). High Kerr nonlinearity of water in THz spectral range. Optics Express.

[40] Balakin Alexei V., Garnov Sergey V., Makarov Vladimir A., Kuzechkin Nikolay A., Obraztsov Petr A., Solyankin Peter M., Shkurinov Alexander P., Zhu Yiming (2018). “Terhune-like” transformation of the terahertz polarization ellipse “mutually induced” by three-wave joint propagation in liquid. Optics Letters.

[41] Vicario, C. *et al*. Intense, carrier frequency and bandwidth tunable quasi single-cycle pulses from an organic emitter covering the terahertz frequency gap. *Sci. Reports* **5**, 10.1038/srep14394 (2015). 10.1038/srep14394 PMC4585874 26400005

[42] Kislin DA, Knyazev MA, Shpolyanskii YA, Kozlov SA (2018). Self-action of nonparaxial few-cycle optical waves in dielectric media. JETP Lett..

[43] Tapia, A. G., Yamamoto, N., Ponseca, C. S. & Tominaga, K. Charge carrier dynamics of znse by optical-pump terahertzprobe spectroscopy. *2011 Int. Conf. on Infrared, Millimeter, Terahertz Waves* 1–2 (2011).

